# The long-term effects of a family based economic empowerment intervention (Suubi+Adherence) on suppression of HIV viral loads among adolescents living with HIV in southern Uganda: Findings from 5-year cluster randomized trial

**DOI:** 10.1371/journal.pone.0228370

**Published:** 2020-02-10

**Authors:** Fred M. Ssewamala, Darejan Dvalishvili, Claude A. Mellins, Elvin H. Geng, Fredderick Makumbi, Torsten B. Neilands, Mary McKay, Christopher Damulira, Proscovia Nabunya, Ozge Sensoy Bahar, Gertrude Nakigozi, Godfrey Kigozi, William Byansi, Miriam Mukasa, Flavia Namuwonge

**Affiliations:** 1 Washington University School of Medicine, Washington University in St. Louis, St. Louis, MO, United States of America; 2 International Center for Child Health and Development (ICHAD), Brown School, Washington University in St. Louis, St. Louis, MO, United States of America; 3 SMART Africa Center, Brown School, Washington University in St. Louis, St. Louis, MO, United States of America; 4 Department of Psychiatry, New York State Psychiatric Institute, HIV Center for Clinical and Behavioral Studies at Columbia University Medical Center, The City of New York, NY, United States of America; 5 Division of Infectious Diseases, John T. Milliken Department of Internal Medicine, Washington University in St. Louis, St. Louis, MO, United States of America; 6 School of Public Health, Makerere University, Kampala, Uganda; 7 Division of Prevention Science, Center for AIDS Prevention Studies (CAPS), Department of Medicine, University of California, San Francisco, San Francisco, CA, United States of America; 8 International Center for Child Health and Development (ICHAD), Uganda Office, Masaka, Uganda; 9 Rakai Health Sciences Program, Kalisizo, Uganda; University of New South Wales, AUSTRALIA

## Abstract

**Background:**

The rapid scale-up of HIV therapy across Africa has failed to adequately engage adolescents living with HIV (ALWHIV). Retention and viral suppression for this group (ALWHIV) is 50% lower than for adults. Indeed, on the African continent, HIV remains the single leading cause of mortality among adolescents. Strategies tailored to the unqiue developmental and social vulnerabilities of this group are urgently needed to enhance successful treatment.

**Methods:**

We carried out a five-year longitudinal cluster randomized trial (ClinicalTrials.gov ID: NCT01790373) with adolescents living with HIV (ALWHIV) ages 10 to 16 years clustered at health care clinics to test the effect of a family economic empowerment (EE) intervention on viral suppression in five districuts in Uganda. In total, 39 accredited health care clinics from study districts with existing procedures tailored to adolescent adherence were eligible to participate in the trial. We used data from 288 youth with detectable HIV viral loads (VL) at baseline (158 –intervention group from 20 clinics, 130 –non-intervention group from 19 clinics). The primary end point was undetectable plasma HIV RNA levels, defined as < 40 copies/ml. We used Kaplan-Meier (KM) analysis and Cox proportional hazard models to estimate intervention effects.

**Findings:**

The Kaplan-Meier (KM) analysis indicated that an incidence of undetectable VL (0.254) was significantly higher in the intervention condition compared to 0.173 (in non-intervention arm) translated into incidence rate ratio of 1.468 (CI: 1.064–2.038), p = 0.008. Cox regression results showed that along with the family-based EE intervention (adj. HR = 1.446, CI: 1.073–1.949, p = 0.015), higher number of medications per day had significant positive effects on the viral suppression (adj.HR = 1.852, CI: 1.275–2.690, p = 0.001).

**Interpretation:**

A family economic empowerment intervention improved treatment success for ALWHIV in Uganda. Analyses of cost effectiveness and scalability are needed to advance incorporation of this intervention into routine practice in low and middle-income countries.

## Introduction

The public health response to adolescents and young persons living with HIV (ALWHIV) in Africa has fallen short, and strategies to engage this group through addressing their unique developmental needs as well as social and structural barriers to care are urgently needed. Over the last ten years, over 20 million persons in resource-limited settings have newly started life-saving ART, but progress has been uneven [1]. Retention and viral suppression among ALWHIV is 50% lower than for adults and while HIV related mortality has fallen by 50% since 2005, HIV remains the single biggest cuase of mortality in adolescents in Africa [2, 3]. Sub-optimal HIV treatment programs for adolescent are, in part, due to the fact that ALWHIV face unique and diverse barriers to retention. They do not control household finances, and therefore, depend on caregivers for access to care. Adolescence is a period of life when peer influence, and therefore risk-taking, stigma and social factors may be particularly powerful [4]. Navigating health systems designed for either younger children or older adults, in particular when coordination with schools is poor, poses distinct challenges to engagement in care [5].

While adolescents face diverse barriers to care, poverty represents an underlying structural factor that exacerbates, either directly or indirectly, many, if not all, of the barriers to engagement in care. In low- and middle-income countries (LMICs), consistent care demands a significant portion of their household income for medication fees, transport to medical visits, and opportunity costs of time lost from work [6]. In quantitative surveys, commonly cited reasons for poor adherence to ART include financial instability, poverty [7] and food insecurity, [8] limited access to medical resources [7], and costs of transportation [9,10]. Adolescents are particularly vulnerable to household poverty, yet adherence to treatment regimens requires a level of economic stability that many poor youths in Africa do not have/experience [15,16]. In short, impoverished children and youth have been found to experience greater challenges to ART adherence compared to children who are more economically stable [9–16]. Yet, to date, no adherence interventions have focused on the underlying economic drivers, which might help explain why results of adherence interventions with HIV positive adolescents and adults living in poverty have had small to moderate effects at best [17,18].

A study by Tuller and colleagues in western Uganda found that provision of free ART without addressing other financial barriers (including the cost of transportation to clinics to pick-up monthly refills) does not sufficiently address the problem of treatment interruptions [9]. The cost of transportation relative to income can be substantial, and often competes with other essential expenses. Individuals who missed medication doses cited problems finding transportation money as a key reason for not being able to maintain their regimen, explaining that they were unable to afford to travel to the clinic before their supply of medication ran out. Even for those not yet on ART, anxiety over the cost of transportation caused them to question whether they would be able to adhere to their medication regimens once they initiated treatment [9]. As a result, HIV positive patients tend to sacrifice healthcare, including adherence to treatment, and other basic needs, including food and school fees for children due to financial constraints [15–18]. Despite the limited literature on economic empowerment (EE) and adolescent adherence, existing research suggests that EE has the potential to significantly improve adherence outcomes, particularly viral suppression [15,16, 19–23].

In order to address the gaps in research, this paper assessed the long-term impact of a family-based EE intervention on viral suppression as an indicator of adherence and HIV control for ALWHIV. We used viral suppression, dichotomized as < 40 copies/ml (Undetectable) vs > 40 copies/ml (detectable), as a proxy measurement of ART adherence.

In this paper, we report outcomes of a five-year cluster randomized trial to test the effect of household EE on ALWHIV aged 10–16 years in HIV affected communitiesin Uganda on adherence to HIV therapy as measured by viral load suppression (VS), dichotomized as < 40 copies/ml (Undetectable) vs > 40 copies/ml (detectable). The data was collected at five time-points (baseline, 12-, 24-, 36-, and 48- month post-intervention initiation). Grounded in asset theory [24], which posits that family EE and asset ownership have tremendous developmental, psychological and social benefits for individuals and households, the study aimed to evaluate the impact of an innovative family based EE intervention composed of incentivized matched financial savings accounts and microenterprise promotion (hereafter, *Suubi+Adherence Intervention*), versus medical and psychosocial Standard of Care (SOC). Both groups received enhanced information sessions on ART adherence using print cartoons that convey adherence topics in a relatable manner.

## Materials and methods

### Study design

**Fig 1:**
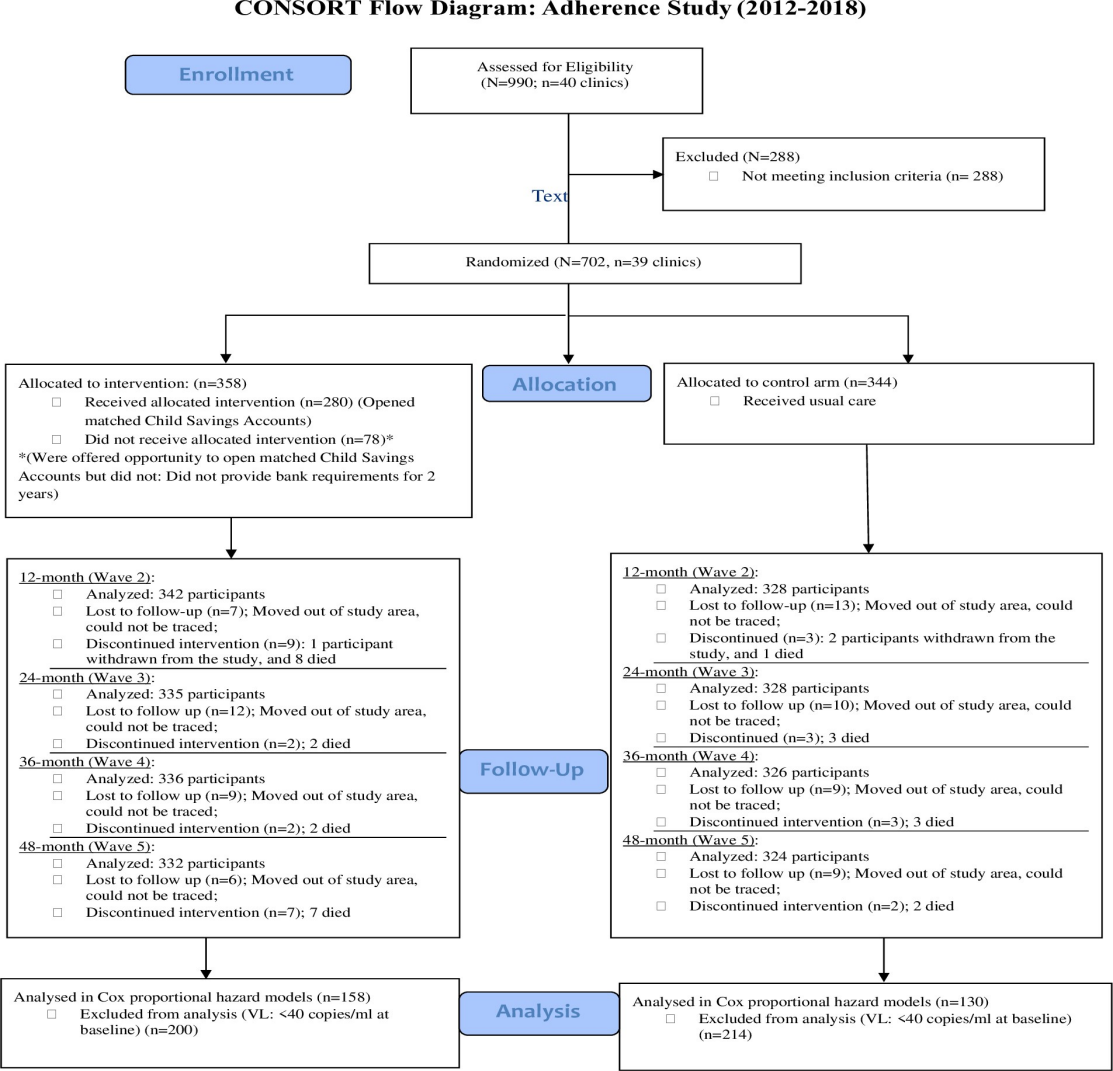
CONSORT flow diagram.

We approached health care facilities in Western Uganda and sought to enroll ALWHIV receiving care at those facilities into the study. Nine-hundred ninety (990) adolescents from 40 health care clinics were identified and screened for eligibility to participate in the study (see Fig 1 for Baseline Consort Flow Diagram). The eligibility criteria for the clinics included: 1) having existing procedures tailored to adolescent adherence (including adolescent clinic days and peer counseling); and 2) being accredited by the Ugandan Ministry of Health as a provider of ART within the five study districts: Rakai, Kyotera, Masaka, Lwengo and Kalungu. Based on those inclusion criteria, one clinic was excluded, leaving a total of 39 clinics within the study sample. The inclusion criteria for adolescents were: 1) 10–16 years at enrollment; 2) HIV-positive and aware of status (previously tested for HIV and confirmed by a medical report); 3) prescribed ART; 4) registered at one of the participating clinics/health centers for follow-up care and drug refills; and 5) non-institutionalized (i.e. living within families and not institutions). Following the screening, consent and assent processes, 702 study participants from the 39 clinics were interviewed by trained staff at baseline. All the interviews were conducted in Luganda—the local language widely spoken in the study area. The remaining 288 adolescents were excluded from study participation due to: a) lack of awareness of HIV status (i.e. not disclosed to by their caregiving families); b) being under or over the study age; and c) in few instances, the inability to comprehend study procedures as assessed during the screening process (Fig 1).

Over the course study, in total, 28 participants died from the disease and 18 participants were lost at subsequent follow-up points (could not be located at subsequent follow-ups and their VL tests could not be performed).

### Ethical clearance

The study obtained IRB approval from Columbia University (Protocol AAAK3852), the Makerere University School of Public Health (Protocol 210) and the Uganda National Council for Science and Technology (Protocol SS 2969). The aims and objectives of the study were explained to both caregivers who provided written consent for their children to participate in the study and their children who provided assent. All research assistants completed certifications in Good Clinical Practices and CITI (Collaborative Institutional Training Initiative) human subjects training. Using back-translation approach/method, all measures were translated into Luganda and back to English by a local expert and certified by Makerere University Institute of Languages (https://llc.mak.ac.ug/departments/deal).

### Randomization

We approached health care facilities in Western Uganda and sought to enroll ALWHIV receiving care at those facilities into the study. Following baseline interviews, 39 clinics were randomly assigned to either the intervention condition (n = 20 clinics, n = 358 participants) or the non-intervention condition (19 clinics, n = 344 participants). The randomization was based on: the number of children served at the health care facility, location—either rural, urban or semi-urban and the level of health facility in Uganda’s health system. For instance, our study included health center III, IVs and hospitals. To balance our randomization, hospitals were randomized separately from other categories. We used a two-arm, cluster randomization design where all participants attending the same clinic were assigned to the same study group. This method was intended to minimize contamination or cross-overs. Randomization of clinics was done by an independent Columbia University Research Assistant.

### The Suubi+Adherence intervention

All participants received medical SOC, as defined by the Uganda Ministry of Health Guidelines for pediatric and adolescent HIV care and treatment; and psychosocial SOC, consisting of information leaflets on adherence and support delivered by lay counselors, including people living with HIV, trained in ART adherence counseling, known as “expert clients”. Due to inconsistency with which SOC is provided in the study region, the Suubi+Adherence study bolstered the SOC in both the control (non-intervention) and the intervention groups with eight information sessions on ART adherence using print cartoons to portray adherence topics in a relatable manner, based on a South African intervention for ALWHIV named VUKA [25]. The bolstered SOC involved eight one-hour group sessions facilitated by a trained research assistant. Adolescents in the intervention group received the bolstered SOC (described above) as well as a family-based EE intervention consisting of child development accounts (incentivized savings accounts matched at a rate of 1:1) plus microenterprise workshops [26]. The incentivized matched savings and microenterprise were for medical expenses and/or education-related expenses for those ALWHIV in school (including school lunches). The microenterprise workshops included four one-hour group sessions intended to provide financial management and microenterprise development training to children and their caregivers. In addition, participants in the treatment condition received 12 group-based educational sessions covering a variety of issues including but not limited to setting goals, business development, and avoiding risk. All components of the financial savings and microenterprise workshops have been previously proven feasible to implement and acceptable to participants [27].

### Statistical analysis

Viral suppression as reflected in HIV VL was used as the primary outcome of this analysis. VL testing was done by the Rakai Health Sciences Program at each of the study’s time points: baseline/pre-intervention initiation (time 0), 12-months post intervention initiation (time 1), 24-months post intervention initiation (time 2), 36-months post intervention initiation (time 3) and 48-months post intervention initiation (time 4). Plasma VL measurements were quantified in blood samples collected in EDTA tubes using Abbott Real Time HIV-1 RNA PCR, version 5.00. VL was dichotomized between undetectable /suppression (VL< 40 copies/ml) and detectable/failed viral suppression (VL≥ 40 copies/ml) levels. In accordance with Abbot Real Time system’s sensivity, 40 copies/ml was the lowest detactable value, and thus the most uniform indicator of viral suppression [28]. This binary indicator of VL, therefore, has greater clinical relevance since most health care practitioners aim for patients to achieve viral suppression.

The goal of any ART adherence is to achieve viral suppression. Thus, for this particular analysis, we used the data of 288 youth who had detectable VL (> = 40 copies/ml) during the baseline testing to explore the impact of the intervention among this group of youth. The primary exposure variable was the family-based EE intervention. In addition, we controlled for demographic factors such as age, sex, schooling status, family structure (orphanhood status, primary caregiver, number of people in the household) and therapy-related factors, such as number of medications (to control the effect of increased pill burden that could lead to decreased adherence) and time since the participant knew his/her HIV status (as an earlier disclosure would be protective) [29, 30], in the models.

Kaplan-Meier analysis was used to determine the cumulative probability of the attainment of undetectable/suppressed VL over the 12, 24, 36 and 48-months. We used the Cox proportional hazard (CPH) model to determine the effect of the intervention on the first time participants had undetectable VL adjusting for participants’ demographic characteristics such as age, sex, orphanhood status, primary caregiver, schooling status as well as the number of medications and the time since they knew their HIV status [29, 30]. The person-time of follow-up was calculated as inter-wave duration between any two subsequent waves. In addition, we conducted marginal models [31] with robust standard errors to adjust for clustering of observations at the clinic level. The CPH model generated the incidence rate ratio as a measure of association with corresponding 95% confidence intervals for all estimates, with < 40 copies/ml coded as the event.

Statistical Significance was determined a priori at the 5% level. All analyses were conducted using STATA version 15.

## Results

### Participant demographics

As presented in Table 1, the majority of study respondents were females (55.21%) with an average age of 12 years. In addition, 26.4% of respondents were double orphans (both birth parents passed) while 35% reported that both parents were still living (non-orphans). About 42% reported a parent as the primary caregiver and 16.7% were not enrolled in school at baseline. The average number of people in the household was 6 adults and 2 children under the age of 18. The average (mean) time since learning of positive HIV status was 4 (95% CI = 3.54–4.21) years.

**Table 1:** Baseline characteristics by study arms (n = 288).

Characteristics	Non-intervention Group n(95% CI)	Intervention Group n(95% CI)	Total n(95% CI)
Overall	130 (45.14)	158(54.86)	288 (100)
**Sex**			
Female	78 (60.00)	81 (51.27)	159 (55.21)
Male	52 (40.00)	77 (48.73)	129 (44.79)
**Age (years) (CI)**			
Age-Mean (CI)	12.39 (12.04–12.74)	12.59(12.27–12.90)	12.5 (12.27–12.73)
**Orpanhood status**			
Double-orphan	38 (29.23)	38 (24.05)	76 (26.39)
Single-orphan	53 (40.77)	58 (36.71)	109 (38.54)
Both parents alive	39 (30.00)	62 (39.24)	103 (35.07)
**Primary Caregiver**			
Parent(s)	47 (36.15)	75 (47.47)	122 (42.36)
Grandparent(s)_	42 (32.30)	44 (27.85)	86 (29.86)
Others	41 (31.54)	39 (24.68)	81 (27.78)
**Current Schooling**			
Not in school	24 (18.46)	24 (15.19)	48 (16.67)
In school	106 (81.15)	134 (84.81)	240 (83.33)
**Number of people in the household**			
Total (2–18) Mean (CI)	5.79 (5.34–6.25)	5.60 (5.21–6.00)	5.69 (5.39–5.99)
Children (0–14): Mean (CI)	2.35 (2.00–2.68)	2.21 (1.89–2.54)	2.27 (2.04–2.51)
**Time since knew HIV status (years)** (CI)			
Mean (CI)	3.97 (3.46–4.48)	3.79(3.34–4.25)	3.87 (3.54–4.21)
**Different types of ART medications**			
One	34 (26.15)	30 (18.99)	64 (22.22)
Two	73 (56.15)	90 (56.96)	163 (56.60)
Three	23 (17.69)	38 (24.05)	61 (21.18)

The attrition rate for these participants across all the 5 waves was 9.7% -22 participants died from the disease, while 6 participants were lost to follow-ups (could not be found at subsequent follow-ups and their VL tests could not be performed). These were not statistically different across study arms (chi2(3) = 4.36, p = 0.23). These participants were censored in corresponding inter-time durations and made 288 at risk at time 0, 271 at risk (17—censored) at time 1; 265 at risk (6-censored) at time 2; 265 at risk (0—censored) at time 3 and 260 at risk (5 –censored) at time 4.

**Fig 2:**
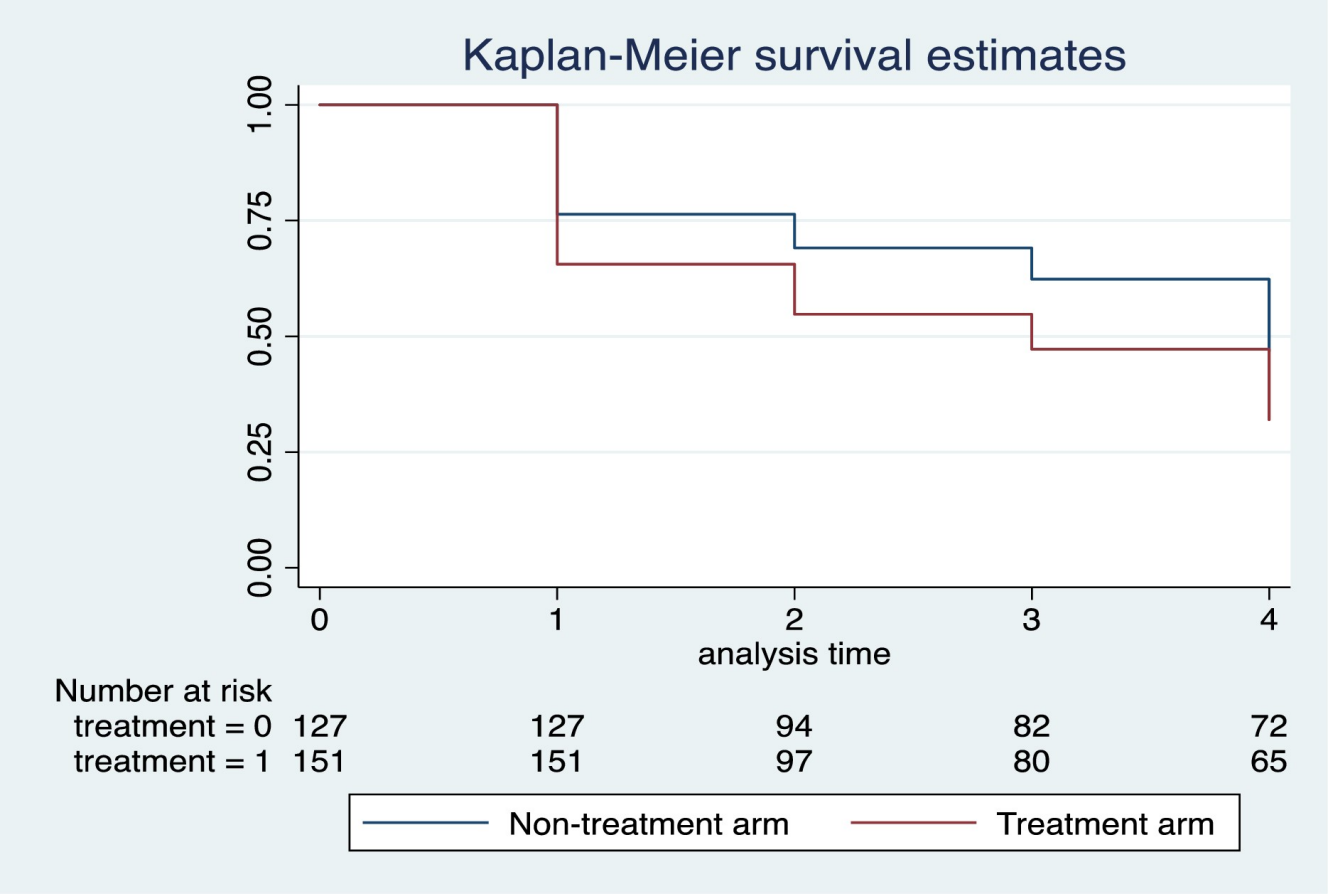
Kaplan-Meier survival estimates.

The Kaplan-Meier results indicate that the total person-years of follow-up were 768 (393 –treatment group, 375 –non-treatment group), resulting in an overall incidence of undetectable VL of 0.21 (165/768); significantly higher in the intervention condition. In total, 165 participants achieved VS (100 participants from intervention group and 65 participants in non-intervention group) (Fig 2). The incident rate of 0.254 (in treatment group) compared to 0.173 (in non-intervention arm) translated into incidence rate ratio of 1.468 (CI: 1.063–2.038), p = 0.008.

The Cox proportional hazard models indicated significant effects of the intervention on participants’ HIV VL detectability. The unadjusted model showed that participants in the intervention group had a higher hazard of 1.445 for undetectable VL (CI: 1.057–1.976, p = 0.021). The results remained significant even after controlling for youth demographics and their treatment specifics. Specifically, the analysis showed that participants in the intervention group had a larger adjusted hazard of undetectable VL (HR = 1.446, CI: 1.073–1.949, p = 0.015). Accounting for sampling variability, the increase in hazard for the intervention group could be as large as 95% or as small as 7% (CI 1.073–1.949). The analysis also points to the fact that the adolescents who were taking two or three different types of ART medications, had a higher hazard for undetectable HIV VL. For instance, in case of two different types of ART medications–the adolescents had 85.2% higher hazard (HR = 1.852, CI: 1.275–2.689, p = 0.001) and in case of three ART medications– 90% higher hazard (HR: 1.90, CI: 1.075–3.362, p = 0.027). (Table 2).

**Table 2:** The results of the adjusted Cox proportional hazard model.

Undetectable viral load (<40 copies/ml)	HR Ratio (95% CI)			p
**Intervention (ref: non-intervention group)**	**1.4457**	**1.0727**	**1.9486**	**0.0155**
**Different types of ART medications (ref: One)**				
Two	**1.8521**	**1.2753**	**2.6896**	**0.0012**
Three	**1.9015**	**1.0754**	**3.3623**	**0.0271**
**Time since knew HIV status (years)**	0.9874	0.9290	1.0495	0.6832
**Sex (ref: male)**	1.1519	0.9260	1.4328	0.2042
**Age (years)**	0.9407	0.8516	1.0390	0.2278
**Current Schooling**	1.2889	0.9379	1.7711	0.1177
**Orpanhood status (ref: both parents are alive)**				
Single orphan	1.2089	0.8530	1.7132	0.2864
Double orphan	1.4857	0.2157	2.7808	0.2157
**Primary Caregiver (ref: Parent(s))**				
Grandparent(s)_	0.6954	0.4127	1.1718	0.1724
Others	0.8345	0.4868	1.4305	0.5106
**Number of people in the household:**				
Adults	0.9914	0.8989	1.0934	0.8629
Children	1.0391	0.8964	1.2046	0.6105
Wald test	chi2(13) = 53.50			
Log pseudolikelihood	-844.30401			
Prob > chi2	<0.0001			
Number of subjects	277			
Number of failures	165			
Time at risk / Number of Observations	767			

Additionally, statistical analyses were conducted to check for proportionality of the model by including time-dependent covariates in the final model [32]. None of the time-dependent variables were significant, either collectively or individually, thus, supporting the assumption of proportional hazard. We checked the model fit using the Cox-Snell residuals and the Nelson-Aalen cumulative hazard function [33]. The hazard function was compared to the diagonal line and observed that hazard function followed the 45-degree line very closely except for very large values of time. Overall, we concluded that the final model fits the data very well. Assessing Schoenfeld residuals also supported the assumption that the model with covariates had proportional-hazards [34].

## Discussion

PLWHIV who adhere to ART treatment are able to live longer and healthier lives [35, 36]. Moreover, when virally suppressed, they are considerably less likely to transmit the virus [37, 38]. Poor or total non-adherence to ART treatment is often attributed to economic reasons, specifically financial instability and poverty [7].

This study examined the impact of a family-based EE intervention on adherence to HIV treatment for ALWHIV using undetectable HIV VL (<40 copies/ml) as a proxy indicator for adherence. Analyses pointed to a significant intervention effect on VL suppression (Table 2) among ALWHIV who had detectable VL at baseline. Results showed that following the initiation of the intervention, the incidence of undetectable VL among participants in the intervention group increased significantly compared to participants in the non-intervention group. This finding supports work by Cluver and colleagues who point to the potential role of economic interventions (e.g. cash transfers) in assisting with HIV care and retention [39].

These findings contribute to the ongoing discussion regarding the need for effective ART to include VL monitoring (both as a health outcome and as a partial proxy measurement of adherence) to ensure that the prescribed ART is working properly to suppress replication of the virus and in case of necessity adjust the treatment accordingly [40, 41]. There is growing concern that unchecked viremia leads to increased odds of HIV transmission, including drug resistance [42]. Testing VL is considered the gold standard for HIV treatment monitoring [43, 44], however, it’s cost and complexity, among other issues, have presented major barriers to its scale-up and use in LMICs [45, 46].

Overall, while our findings point to the potential of family based EE interventions in achieving VL suppression among ALWHIV, there is a need for more research to determine the mechanisms through which the observed change happens For example, the VL suppression could potentially be attributed to associated food security and the ability to access treatment in a timely manner [47–49]. Alternatively, the fact that the intervention brings financial stability to the home may result in increased medication-adherence because the ALWHIV feel more hopeful [50]. Such factors need to be further examined both qualitatively and quantitatively. Thus, research is needed to also explore mediators and moderators of the family-based EE interventions on VL suppression.

## Limitations

There are two main limitations of the study. First, the analyses only included data of youth who had detectable VL at baseline and did not explore the impact of the treatment on those who were undetectable at baseline, but may have become detectable, with possible VL blips (fluctuation of VL throughout the course of the study). Also, as this paper focused on the impact of the treatment only on the dichotomized outcome variable, dichotomized as undetectable (<40 copies/ml) vs detectable (≥40 copies/ml), further research is needed to explore the impact of the treatment on continuous VLs or other cut points.

Second, even though testing VL is considered the gold standard for HIV treatment monitoring and an undetectable VL level is consistent with good medication adherence, detectable levels are not necessarily universally diagnostic of non-adherence, given that other factors may influence viremia, including treatment resistance [42]. In addition, biological markers only provide data for one specific point and do not usually reflect variations in adherence over time [51]. Viral suppression takes time and viremia may also take time to emerge. By the time someone is not virally suppressed, they most probably have been non-adherent for some time [52]. Thus, more regular assessments/check-ups for adherence using different direct and non-direct methods are recommended.

## Conclusions

The study findings indicated that family-based EE interventions can increase rates of VL suppression, and thereby, improve the health of youth, minimize HIV transmission, and superinfections [53]. However, there is a need for more research on the impact of family-based EE interventions on ART adherence in more detail. Specifically, Suubi+Adherence supported children and families to gain financial literacy and strengthen their economic security. Research should be continued to explore the effect of EE interventions on food insecurity, poverty and financial constraints associated with HIV and ART adherence in general.

## Clinical significance

Our findings suggest that a family-based EE intervention has the potential to improve viral suppression, and thus, the health of vulnerable ALWHIV in low-resource environments. These findings contribute to the discussion on ending the HIV/AIDS epidemic and ART responses, and emphasize the need for further research and policy dialogue on the intersections of financial security and HIV prevention and treatment.

## Supporting information

S1 Checklist CONSORT 2010 checklist of information to include when reporting a randomised trial*. (DOC)
